# Supplementary material for: The genome sequence of the White-banded Grass-moth, Crambus pascuella Linnaeus, 1758 (Lepidoptera: Crambidae)
Source: Wellcome Open Res. 2026 Sep 28;11:773. [Version 1] doi: 10.12688/wellcomeopenres.27970.1 (PMC13680838; doi:10.12688/wellcomeopenres.27970.1)
Supplement: Peer review history

# Reviewer report: Reviewer response for version 1

**DOI:** 10.21956/wellcomeopenres.30772.r171830

**Published:** 2026-10-19

**Authors:** Marian R. Goldsmith

**Affiliations:** 1 University of Rhode Island, Kingston, Rhode Island, USA

Laboratory protocols and technical bioinformatic analyses are detailed, comprehensive, and available in the text and on the internet. They are also referred to and published by the founding group and others with numerous sources accessible via internet links and traditional references provided in the text. This information includes software as well as basic material and equipment. The internet links are especially useful considering how fast this field is moving and the possibility that some of this kind of information may be outdated relatively soon and then perhaps difficult to find.

 Further, the authors declare that the project material is used and distributed under specified ethical and legal considerations and practices.

 As a partial summary of the information provided and to provide a “flavor” of the presentation, below are selected excerpts from the text [enclosed in quotes] which I copied directly from the report to highlight the research aims, scope, and findings of the project rather than summarizing the information in my own words.

**Summary of basic approaches**

**[begin excerpted text in quotes]**

 "The haplotype 1 assembly was assessed using BlobToolKit (Challis et al., 2020). PacBio reads were mapped to the assembly to derive sequence coverage, and BUSCO (Manni et al., 2021) was used to assess gene-set completeness. DIAMOND BLASTp searches of extracted BUSCO proteins and DIAMOND BLASTx searches of assembly sequences against UniProt Reference Proteomes (Buchfink et al., 2021), followed by BLASTn searches of sequences without DIAMOND BLASTx hits against the NCBI nt database (Altschul et al., 1990), supported taxonomic assignment. Coverage, sequence composition, taxonomic assignment and BUSCO results were integrated into a BlobDir for interactive inspection and visualisation. The snail and blob plots were rendered locally from this hosted BlobDir using BlobTk v0.8.3. The scaffold-length radial scale function for the snail plot was set to linear (Challis & Blaxter, 2026). The blob plot used circular markers with area proportional to scaffold length (Challis et al., 2020).

 Ancestral linkage groups were painted along the chromosome-level scaffolds of the haplotype 1 assembly. Complete BUSCO genes (single-copy and duplicated) from the lepidoptera_odb10 lineage dataset were assigned to the 32 Lepidoptera Merian elements (MZ and M1–M31) described by Wright et al. (2024) and plotted along chromosomes drawn to scale.

**Sequence data**

 PacBio sequencing of the C. pascuella specimen generated 41.37 Gb (gigabases) from 3.68 million reads. These reads were used to assemble the genome. GenomeScope analysis using a p = 2 model gave a k-mer-based haploid genome size estimate of 777.10 Mb, with heterozygosity of 2.09% and repeat content of 46.39%; the full-model fit was 98.15% (Figure 2). The GenomeScope estimates guided expectations for the assembly. The NCBI assembly record reports 52× genome coverage. Hi-C sequencing produced 144.8 Gb from 479.50 million reads, which were used to scaffold the haplotype 1 assembly. Table 1 summarises the specimen an sequencing details.

**Assembly statistics**

 The **moth** **genome** was assembled into two haplotypes using Hi-C phasing. Both haplotype 1 and haplotype 2 assemblies were curated to chromosome level. The haplotype 1 assembly, which is proposed as the reference assembly for this species, has a total length of 792.17 Mb in 228 scaffolds (excluding organelle sequences), with 164 gaps, and its scaffold N50 is 27.65 Mb (Table 2). The scaffold auN for the haplotype 1 assembly is 28.3 Mb.

**BUSCO analysis** using the lepidoptera_odb10 reference set (n = 5,286) identified 98.6% of the expected gene set (single = 98.2%,duplicated = 0.4%) in the haplotype 1 assembly. For the haplotype 2 assembly, BUSCO v.5.5.0 analysis identified 98.7% of the expected gene set (single = 98.1%, duplicated = 0.6%). Variation in GC, AT, and N content was assessed and reported, in addition to GC proportion an base converage.

 The **mitochondrial genome** was also assembled (length 15.34 kb, OZ205629.1). This sequence is included as a contig in the multifasta file of the haplotype 1 genome submission and as a standalone record." **[end of excerpted text]**

**Additional Reviewer Comments**: Given the aims and scope of the Darwin Tree of Life Project to document genomes of all organisms (or all Lepidoptera in this case) found in Britain and Ireland, the choice of this species is unquestionably valid, relevant, and justified for generating a comprehensive and complete genome sequence. Additionally, as an investigator long interested in the scope and analysis of genome projects for Lepidoptera in particular, and with a possibility to broaden the impact and applications of this research for other scientists and for the general public, I would have liked to read a little more information about specific or special features of this species in addition to its ecologically distinctive habitat and relatively widespread geographic distribution, from Europe to Siberia, Japan, and North America, as noted in the text. As a minor note, this species seems to be more widespread than the subjects of the few other DTOL Lepidoptera genome project reports I read for this review which are found only in Britain and Ireland, and perhaps argues for a wider significance of this species. For example, based on this moth’s taxonomic assignment to the Crambidae, its genome may provide a resource to define and compare the genomes of relatively closely related stem and stalk borers which are serious pests of important food crops such as rice, millet, corn, bean pod, and sugar cane [https://en.wikipedia.org/wiki/Crambidae]. Thus, having this first complete genome sequence for such a close relative could provide an important genomic resource for developing new and innovative environmentally safe ways to control its brethren.

Are the datasets clearly presented in a usable and accessible format, and the assembly and annotation available in an appropriate subject-specific repository?

Yes

Are sufficient details of the sequencing and extraction, software used, and materials provided to allow replication by others?

Yes

Are the rationale for sequencing the genome and the species significance clearly described?

Yes

Are the protocols appropriate and is the work technically sound?

Yes

Reviewer Expertise:

Lepidopteran genetics and genomics

I confirm that I have read this submission and believe that I have an appropriate level of expertise to confirm that it is of an acceptable scientific standard.

# Reviewer report: Reviewer response for version 1

**Published:** 2026-09-28

**Authors:** Automated Benchmarking

**Affiliations:** 1 Wellcome Sanger Institute, Hinxton, UK

As this article is a Genome Note, Automated Benchmarking has been used to determine whether the reported assembly passes four defined checks established by the Earth BioGenome Project: the raw and assembled data are publicly available, the Earth BioGenome Project three-part metric is met, the k-mer completeness exceeds 90%, and the BUSCO score passes the stated threshold. For Crambus pascuella (ilCraPasc1), all four checks passed. Data available: pass. ENA study PRJEB82173, raw-read accessions ERR13946464 and ERR13947484, and assembly accessions GCA_964341525.1 and GCA_964341515.1 are public. Earth BioGenome Project three-part metric: pass. Haplotype 1 is reported as 6.C.Q63, meeting the 6.C.Q40 reference standard. k-mer completeness: pass. The combined haplotype assemblies have 99.42% k-mer completeness, exceeding the Earth BioGenome Project target of 90%. BUSCO: pass. Haplotype 1, which is proposed as the reference assembly for this species, has 98.6% complete BUSCOs (98.2% single-copy and 0.4% duplicated). BUSCO software version: 5.5.0; lineage dataset: lepidoptera_odb10. It meets the target of greater than 90% complete and single-copy BUSCOs. This decision uses values reported in the Genome Note and public accession records.

Reviewer Expertise:

NA

I confirm that I have read this submission and believe that I have an appropriate level of expertise to confirm that it is of an acceptable scientific standard.
